# Unresolved Recombination Intermediates Cause a *RAD9*-Dependent Cell Cycle Arrest in *Saccharomyces cerevisiae*

**DOI:** 10.1534/genetics.119.302632

**Published:** 2019-09-27

**Authors:** Hardeep Kaur, Krishnaprasad GN, Michael Lichten

**Affiliations:** Laboratory of Biochemistry and Molecular Biology, Center for Cancer Research, National Cancer Institute, Bethesda, Maryland 20892

**Keywords:** double Holliday junction dissolution, Sgs1-Top3-Rmi1 helicase-decatenase, DNA damage checkpoint, mitotic cell cycle, homologous recombination

## Abstract

In *Saccharomyces cerevisiae*, the conserved Sgs1-Top3-Rmi1 helicase-decatenase regulates homologous recombination by limiting accumulation of recombination intermediates that are crossover precursors. *In vitro* studies have suggested that this may be due to dissolution of double-Holliday junction joint molecules by Sgs1-driven convergent junction migration and Top3-Rmi1 mediated strand decatenation. To ask whether dissolution occurs *in vivo*, we conditionally depleted Sgs1 and/or Rmi1 during return to growth (RTG), a procedure where recombination intermediates formed during meiosis are resolved when cells resume the mitotic cell cycle. Sgs1 depletion during RTG delayed joint molecule resolution, but, ultimately, most were resolved and cells divided normally. In contrast, Rmi1 depletion resulted in delayed and incomplete joint molecule resolution, and most cells did not divide. *rad9***∆ mutation restored cell division in Rmi1-depleted cells, indicating that the DNA damage checkpoint caused this cell cycle arrest. Restored cell division in Rmi1-depleted *rad9***∆ cells frequently produced anucleate cells, consistent with the suggestion that persistent recombination intermediates prevented chromosome segregation. Our findings indicate that Sgs1-Top3-Rmi1 acts *in vivo*, as it does *in vitro*, to promote recombination intermediate resolution by dissolution. They also indicate that, in the absence of Top3-Rmi1 activity, unresolved recombination intermediates persist and activate the DNA damage response, which is usually thought to be activated by much earlier DNA damage-associated lesions.

## Abstract (precis)

It has been suggested that the conserved Sgs1-Top3-Rmi1 (STR) helicasedecatenase complex resolves double Holliday junction recombination intermediates (dHJs) as noncrossovers by a process called dissolution. Lichten, Kaur, and GN tested this by accumulating dHJs during meiosis...

THE conserved STR/BTR complex, which contains the RecQ-family helicase Sgs1 (BLM in many organisms), topoisomerase III (Top3, Top3α in mammals), and RecQ-mediated genome instability protein 1 (Rmi1, BLAP75 in humans), has important functions that maintain genome integrity (Bernstein *et al.* 2010; Larsen and Hickson 2013; Crickard and Greene 2019). STR complex components have two principal biochemical activities: Sgs1/BLM is a 3′ to 5′ helicase that unwinds DNA (Bennett *et al.* 1998; Chu and Hickson 2009; Bernstein *et al.* 2010), and Top3-Rmi1 has robust single-strand DNA passage but weak supercoil relaxing activities (Cejka *et al.* 2012). *In vitro*, STR/BTR has activities that can both promote and limit homologous recombination. Sgs1 and the Dna2 nuclease catalyze DNA end-resection, producing 3′-ended single-strand DNA that invades homologous duplex sequences to initiate homologous recombination; this activity is stimulated by Top3-Rmi1 (Gravel *et al.* 2008; Zhu *et al.* 2008; Cejka *et al.* 2010; reviewed in Mimitou and Symington 2009). Other STR/BTR activities have the potential to limit the formation of crossover (CO) recombinants (Figure 1). STR/BTR disassembles D-loop structures that are analogs of initial strand invasion products (van Brabant *et al.* 2000; Bachrati *et al.* 2006; Fasching *et al.* 2015). This directs events toward a process called synthesis-dependent strand annealing (SDSA) that produces noncrossover (NCO) recombinants (Gloor *et al.* 1991), and prevents formation of the double Holliday junction joint molecules (dHJ-JMs) that are potential CO precursors (Szostak *et al.* 1983). STR/BTR also has an *in vitro* activity, called dissolution, that takes apart dHJ-JMs and produce NCOs via helicase-driven convergent HJ migration coupled with Top3-Rmi1-catalyzed strand passage (Wu and Hickson 2003; Plank *et al.* 2006; Wu *et al.* 2006). These two activities can have different consequences. D-loop disassembly can redirect events to different recombination pathways, since taking apart an early intermediate recreates a lesion that can undergo additional rounds of invasion and disassembly (De Muyt *et al.* 2012; Kaur *et al.* 2015; Piazza and Heyer 2019). In contrast, dHJ dissolution directly produces a mature NCO, and thus terminates the recombination process.

**Figure 1:**
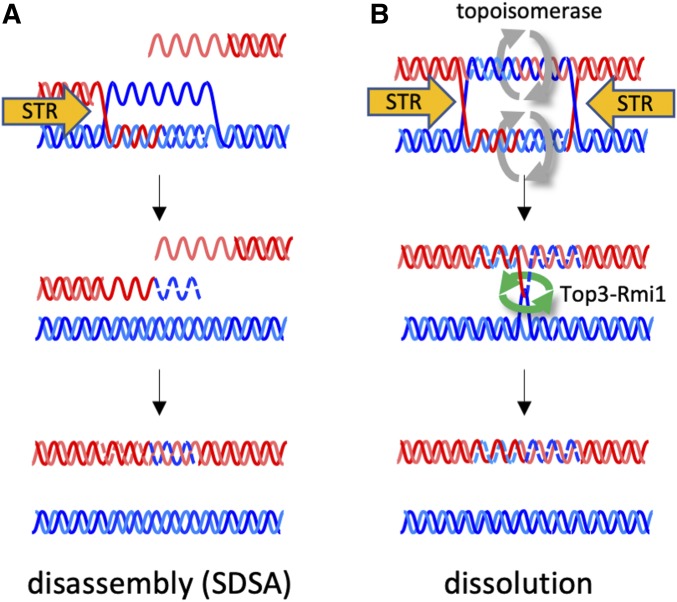
Two possible Sgs1-Top3-Rmi1 anti-crossover activities. (A) D-loop disassembly and synthesis-dependent strand annealing (SDSA). Following strand invasion and 3′ end-primed synthesis (indicated by dashed lines), an unwinding activity (orange arrow, in this case the STR complex) takes apart a D-loop, releasing the invading break end. Annealing with the other break end, followed by gap-filling synthesis, produces a noncrossover (NCO) recombinant. (B) Dissolution. Unwinding activities drive convergent Holiday junction migration, facilitated by relief of overwinding (gray arrows), to produce two linear DNA molecules linked by at least one hemicatenane. Single-strand passage by Top3-Rmi1 (green arrows) resolves hemicatenanes and produces a NCO recombinant.

Consistent with these *in vitro* activities, *sgs1*, *top3*, and *rmi1* mutants (hereafter referred to collectively as *str* mutants) are DNA damage-sensitive, display elevated levels of mitotic crossing-over, and show synthetic lethality with mutants lacking the Mus81-Mms4 or Slx1-Slx4 nucleases that resolve dHJ-JMs (Wallis *et al.* 1989; Mullen *et al.* 2001, 2005; Shor *et al.* 2002; Ira *et al.* 2003; Ehmsen and Heyer 2008; Wyatt and West 2014). This synthetic lethality is suppressed by reducing homologous recombination (Fabre *et al.* 2002), suggesting that *str* mutants accumulate recombination intermediates that are toxic if unresolved. *str* mutants also accumulate increased levels of DNA damage-induced JMs relative to wild type (Ashton *et al.* 2011; Mankouri *et al.* 2011)—again consistent with a role for STR in limiting dHJ-JM accumulation.

The Top3-Rmi1 heterodimer also has important activities independent of Sgs1. Cells lacking Top3 or Rmi1, but not Sgs1, display slow growth, persistent signals of DNA damage, and accumulate cells at G2/M, consistent with low-level induction of the DNA damage response (Wallis *et al.* 1989; Gangloff *et al.* 1994; Chakraverty *et al.* 2001; Chang *et al.* 2005; Mullen *et al.* 2005). Loss of either Sgs1 or homologous recombination suppresses these phenotypes (Gangloff *et al.* 1994; Oakley *et al.* 2002; Chang *et al.* 2005; Shor *et al.* 2005), suggesting that Top3-Rmi1 limits the accumulation of toxic recombination intermediates formed by Sgs1. The Top3-Rmi1 heterodimer also has Sgs1-independent functions during meiosis. In *top3* and *rmi1* mutants, but not in *sgs1*, a substantial fraction of JMs remain unresolved; while these unresolved JMs do not affect the timing of the two meiotic nuclear divisions, they do impair chromosome segregation (Kaur *et al.* 2015; Tang *et al.* 2015). This indicates that Top3-Rmi1 prevents the accumulation of JMs where the two parental DNA molecules are linked by structures, such as hemi-catenanes (Giannattasio *et al.* 2014), which are not resolved by the structure-selective nucleases (SSNs) Mus81-Mms4, Yen1, and Slx1-Slx4 (Kaur *et al.* 2015; Tang *et al.* 2015). Interestingly, unlike in mitotic cells, *sgs1* mutation does not suppress the meiotic JM-resolution defect of *top3* and *rmi1* mutants, indicating that Sgs1 does not form these unresolvable intermediates.

While these findings point to an important role for STR in modulating homologous recombination, they do not distinguish between D-loop disassembly and dHJ-JM dissolution. Support for D-loop disassembly has come from studies of meiotic and mitotic recombination. In budding yeast, most meiotic NCOs are thought to be formed by SDSA, without a stable dHJ-JM intermediate, while most meiotic COs derive from dHJ-JMs (Allers and Lichten 2001). These JMs are stabilized by an ensemble of meiosis-specific proteins called the ZMM proteins (Börner *et al.* 2004; Lynn *et al.* 2007; Pyatnitskaya *et al.* 2019) and are resolved as COs by the MutLγ (Mlh1, Mlh3, Exo1) complex (Argueso *et al.* 2004; Zakharyevich *et al.* 2010; 2012). Consistent with STR-mediated D-loop disassembly, *str* mutants no longer form meiotic NCOs by SDSA, and, instead, all events form ZMM-independent JMs that are resolved by SSNs that also resolve JMs during the mitotic cell cycle (Oh *et al.* 2007; Matos *et al.* 2011; De Muyt *et al.* 2012; Kaur *et al.* 2015; Tang *et al.* 2015). Evidence that STR activity limits strand-invasion intermediate formation in mitotic cells is provided by studies that used a proximity ligation assay to detect early chromosome associations during DSB repair (Piazza *et al.* 2019); this signal increased about twofold both in sgs1∆ mutants and in strains overexpressing a catalysis-dead *top3* mutant protein. Because this study used a repair substrate with homology only to one side of the DSB, it could not directly address the role of the STR complex in modulating dHJ-JM formation.

*In vivo* data supporting dissolution has been even more limited, and has come from studies using yeast *ndt80* mutants, which remain in meiosis I prophase and do not resolve JMs. Tang *et al.* (2015) combined conditional-depletion allele of *RMI1* with inducible expression of *NTD80* to study JM resolution under conditions of Rmi1 depletion. Rmi1-depleted cells displayed incomplete JM resolution and chromosome segregation defects, consistent with a role for Top3-Rmi1 in resolving at least some of the JMs that form during normal meiosis. Due to ongoing JM formation during the initial stages of Rmi1-depletion, this study could not exclude the possibility that JMs with altered structures were formed under conditions of reduced STR activity, and that these JMs were responsible for the observed resolution and chromosome segregation failures.

In a second study, Dayani *et al.* (2011) examined meiotic JM resolution during return-to-growth (RTG). In RTG, cells undergoing meiosis are shifted to rich growth medium, whereupon they exit meiosis and return to the mitotic cell cycle, during which time meiotic JMs are resolved under conditions similar to the G2 phase of the mitotic cell cycle (reviewed in Simchen 2009). Consistent with STR promoting early JM resolution by dissolution, JM resolution during RTG was delayed, relative to wild type, in substrate recognition-defective sgs1-∆*C795* mutants (Schiller *et al.* 2014). However, this study could not exclude the possibility that JMs with altered structures form during meiosis in sgs1-∆*C795* mutants, and that this structural difference, rather than the absence of active Sgs1, was responsible for the observed delay in resolution.

To further test STR complex-mediated dHJ-JM dissolution *in vivo*, we used an experimental approach that combines RTG with conditional depletion of Sgs1 and/or Rmi1, so that JMs formed during meiosis in the presence of normal STR function could then be resolved during RTG in either the presence or absence of active STR. Our findings support a role for STR-mediated JM resolution by dissolution during the mitotic cell cycle, and provide further evidence for an Sgs1-independent Top3-Rmi1 function during JM resolution. In addition, we provide evidence that the DNA damage response prevents cell cycle progression when unresolved recombination intermediates are present.

## Materials and Methods

### Strains

Yeast strains (Table 1) are derived from the haploid parents of MJL2984 (Jessop *et al.* 2005) by genetic crosses or transformation, and are of the SK1 background (Kane and Roth 1974). Transformants were confirmed by PCR and/or Southern blot analysis. All protein fusions were confirmed by sequencing PCR products amplified from the genome.

**Table 1:** Strains

Name	Genotype
MJL3807	*URA3*::*Ptef1-OsTIR1/ URA3*::*Ptef1-OsTIR1 SGS1-3xHA-IAA17-hygMX/SGS1-3xHA-IAA17-hygMX*
MJL3847	*URA3*::*Pzeo1-OsTIR1/ URA3*::Pzeo1-OsTIR1 RMI1-AID*-9xMYC-hphNT1/ RMI1-AID*-9xMYC-hphNT1
MJL3863	*URA3*::*Pzeo1-OsTIR1/ URA3*::Pzeo1-OsTIR1 SGS1-3xHA-IAA17-hygMX/SGS1-3xHA-IAA17-hygMX RMI1-AID*-9xMYC-hphNT1/ RMI1-AID*-9xMYC-hphNT1
MJL3899	*URA3*::*Pzeo1-OsTIR1/ URA3*::Pzeo1-OsTIR1 RMI1-AID*-9xMYC-hphNT1/ RMI1-AID*-9xMYC-hphNT1 PDS1-18xMYC-LEU2/ PDS1-18xMYC-LEU2
S5338xS5344^a^	*URA3*::Pzeo1-OsTIR1/ ura3 RMI1-AID*-9xMYC-hphNT1/ RMI1-AID*-9xMYC-hphNT1 PDS1-18xMYC-LEU2/ PDS1-18xMYC-LEU2 mad1∆::*natMX/mad1∆*::*natMX*
S5342xS5348^a^	*URA3*::Pzeo1-OsTIR1/ ura3 RMI1-AID*-9xMYC-hphNT1/ RMI1-AID*-9xMYC-hphNT1 PDS1-18xMYC-LEU2/ PDS1-18xMYC-LEU2 rad9∆::*natMX/rad9*::*natMX*

All strains are *MAT***a***/MATα*, are homozygous for *lys2* *ho*::*LYS2* *arg4*∆*(Eco47III-HpaI) ndt80*∆*(Eco47III-BseRI)*::*kanMX6*, and contain the inserts (*his4*::*URA3*-*arg4-ecPal9* *leu2*-*R/HIS4* *leu2-R*::*URA3*-*ARG4*) illustrated in Figure S1, available at https://doi.org/10.25386/genetics.9841316. *URA3*::*Ptef1-OsTIR1* and *URA3*::*Pzeo1-OsTIR1* contain a codon-optimized rice auxin-responsive F-box protein (Kubota *et al.* 2013) transcribed from a highly-expressed, constitutive promoter (Kaur *et al.* 2018). *SGS1*-*3xHA-IAA17* contains three HA epitopes followed by the IAA17 auxin-dependent degron (Nishimura *et al.* 2009). *RMI1*-AID*-9xMYC (Tang *et al.* 2015) contains a truncated IAA17 (amino acids 71–114) followed by nine MYC epitopes (Morawska and Ulrich 2013). For simplicity, these constructs are referred to as Sgs1-AID and Rmi1-AID, respectively.

a Fresh diploids of these strains were isolated before each experiment.

### Return to growth

Induction of meiosis, RTG, and protein depletion were as described (Dayani *et al.* 2011; Kaur *et al.* 2018). Briefly, meiosis was induced in 400 ml liquid cultures at 30°. After 6 hr, cells were harvested by centrifugation, washed with water, resuspended in the same volume of growth medium (YPAD) prewarmed to 30°, divided equally between two 2-liter baffled Erlenmeyer flasks and aerated vigorously (350 rpm) at 30°. Auxin (indole acetic acid, 0.5 M stock in DMSO) was added to one culture to a final concentration of 2 mM, and the same volume of DMSO was added to the other culture. These additions were repeated every subsequent hour. Samples for DNA, protein, and cytological analysis were collected at indicated time points.

### DNA extraction and analysis

DNA isolation and recombination intermediate and product detection were performed as described (Allers and Lichten 2000, 2001; Oh *et al.* 2009). As illustrated in Supplemental Material, Figure S1, available at https://doi.org/10.25386/genetics.9841316, recombination intermediates were scored on blots of gels containing *Xmn*I digests, probed with *ARG4* coding sequences (+156 to +1413). CO and NCO products were scored on blots of gels containing *Eco*RI–*Xho*I digests, probed with *HIS4* coding sequences (+539 to +719).

#### Protein extraction and western blotting:

Protein extracts were made by TCA precipitation (Foiani *et al.* 1994) from 3 ml of culture. Gel electrophoresis, blotting, and probing were performed as described (Kaur *et al.* 2018). Primary antisera and dilutions: mouse anti-HA monoclonal (clone 12CA5, 11583816001; Roche), 1/10,000; rabbit anti-MYC (Santa Cruz Biotechnology, sc-789), 1/1000; goat anti-ARP7 (Santa Cruz Biotechnology, sc-8961), 1/1000. Secondary antibodies were alkaline phosphatase conjugates of goat anti-mouse IgG (A3562; Sigma); rabbit anti-goat IgG (A4187; Sigma); and goat anti-rabbit IgG (A3687; Sigma). All were used at 1/10,000 dilution.

#### Cytology:

Cells were prepared for immunostaining as described (Xaver *et al.* 2013) with the following modifications. Cells were fixed with three successive incubations (10–15 min, room temperature) in 3.4% formaldehyde, the latter two in 0.1 M potassium phosphate, 0.5 mM MgCl_2_, pH 6.4, and then stored at 4°. Spheroplasting used 0.5 mg/ml Zymolyase 100T (Nacalai USA #07655) in place of Zymolyase 20T. Slides were immunostained overnight at 4° or 4 hr at 30° with a mixture of the two primary antisera diluted in blocking buffer [rat anti-tubulin (ab6160 1:1250; Abcam) and rabbit anti-MYC (sc-789 1:250; Santa Cruz)], washed in PBS (three times, 5 min, room temperature), and then incubated with secondary antisera [Cy3-conjugated donkey anti-rabbit IgG (#711-165-152; Jackson Laboratories) and FITC-conjugated rabbit anti-rat IgG (# F1763; Sigma), both 1:600 in blocking buffer] for 3hr at 30°, followed by three 5-min room temperature washes in PBS. Samples to be examined by DAPI-staining only were treated as described (Goyon and Lichten 1993) after formaldehyde fixation and storage as above.

#### Estimation of unresolved joint molecules:

**Figure 2:**
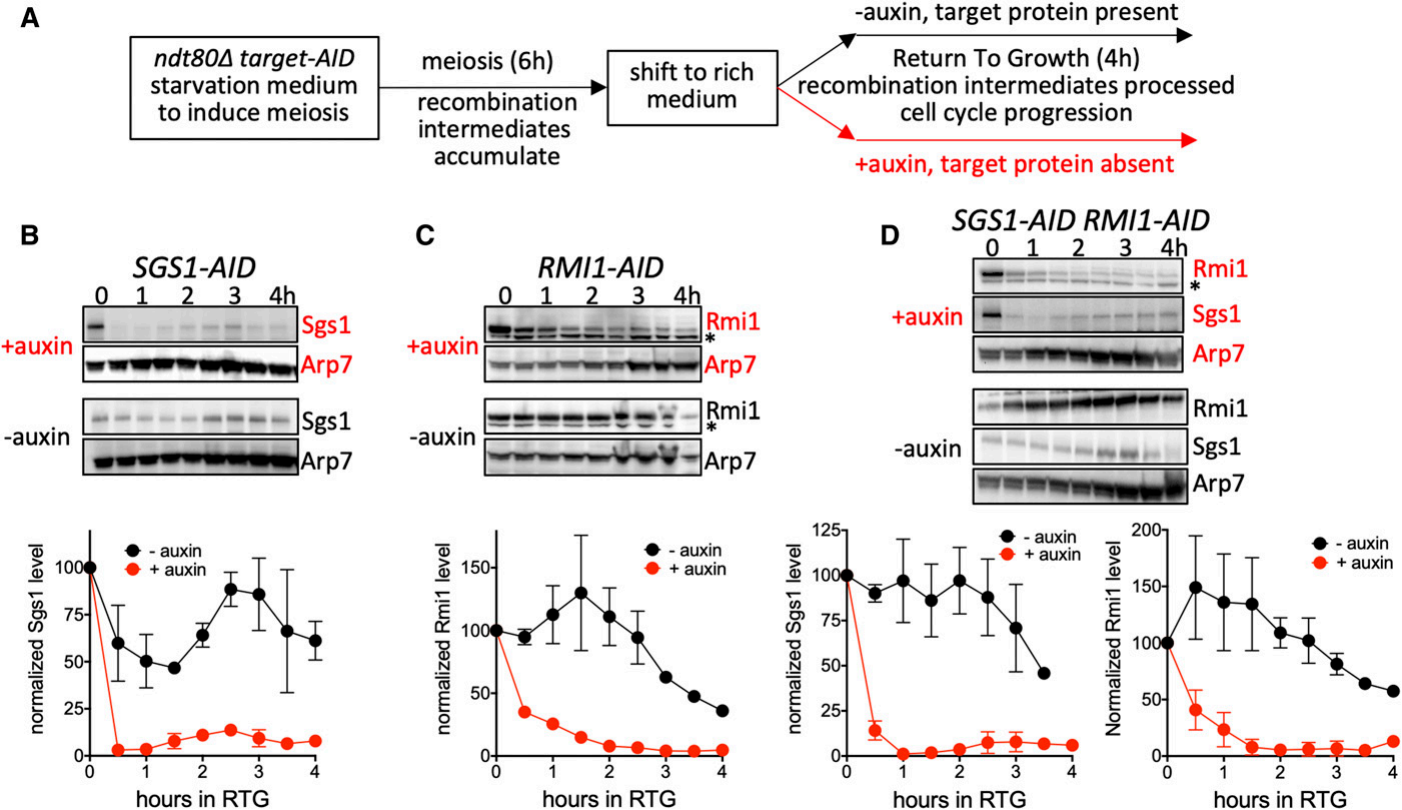
Use of auxin-inducible degrons during return to growth (RTG). (A) Experimental plan. Diploid budding yeast *ndt80*∆ mutants, containing an auxin-inducible degron (AID) fused to the protein of interest and constitutively expressing the rice OsTIR1 auxin response F-box protein, are induced to undergo meiosis and accumulate meiotic recombination intermediates for 6 hr. Cells are then shifted to rich medium, at which point they re-enter the mitotic cell cycle, during which cell cycle landmarks, recombination intermediates, and products are monitored. If auxin is present, the protein of interest is degraded; if auxin is absent, the protein of interest remains. (B) Auxin induced degradation of Sgs1-AID (MJL3807). Top: representative Western blot sections, probed with anti-HA to detect Sgs1 and with anti-Arp7 as a loading control. Times are hours after shift to rich medium. Bottom: normalized Sgs1 levels (Sgs1/Arp7, with 0 hr set to 100); red, auxin present; black, vehicle only. (C) Auxin-induced degradation of Rmi1 (MJL3847), details as in (B), except that anti-Myc was used to detect Rmi1. The lower band in the Rmi1 panels (*) is a cross-reacting protein. (D) Auxin-induced degradation of Sgs1 and Rmi1 in a doubly tagged strain (MJL3863), details as in (B and C). Values are the mean of two independent experiments; error bars indicate range.

The number of unresolved JMs in Rmi1-depleted cells were estimated, starting with previous calculations of ∼90 interhomolog COs per nucleus (Chen *et al.* 2008; Mancera *et al.* 2008; Martini *et al.* 2011), and assuming a 1:1 correspondence between COs and JMs. Intersister JMs are present at ∼1/4 the level of interhomolog JMs (Goldfarb and Lichten 2010). Therefore, we estimate the total of JMs formed per cell during meiosis to be 90 (interhomolog) + 22.5 (intersister). In Rmi1-depleted cells, ∼20% of JMs remain unresolved at 4 hr after return to growth (Figure 2B, below). This corresponds to ∼18 interhomolog JMs and 4.5 intersister JMs remaining unresolved. The first nuclear division after RTG involves sister chromatid segregation (Dayani *et al.* 2011), so all of the unresolved intersister JMs are expected to prevent sister chromatid segregation. We presume random segregation of homolog chromatids; a given pair of homolog chromatids should segregate to the same pole in half of the cells, and to opposite poles in half of the cells, so 1/2 of all unresolved interhomolog JMs are expected to prevent chromatid segregation during the first division after RTG. Therefore, we estimate that there will be 9 unresolved interhomolog JMs per nucleus and 4.5 unresolved intersister JMs per nucleus in a configuration that will prevent chromosome segregation during the first division after RTG. Because cells undergoing RTG are tetraploid, with 32 chromatids pairs (Dayani *et al.* 2011), this corresponds to ∼40% of all chromatids.

#### Data availability:

All experimental materials not supplied commercially will be supplied upon request. Authors affirm that all data necessary to confirm the conclusions of this article are present within the article, figures and tables. Numerical values underlying graphs in all figures are provided in File S1. Supplemental material available at FigShare: https://doi.org/10.25386/genetics.9841316.

## Results

### Targeted degradation of Sgs1 and Rmi1 during RTG

To study STR function during RTG, we used auxin-mediated protein degradation (Nishimura *et al.* 2009; Kaur *et al.* 2018) to deplete Sgs1 and/or Rmi1 (Figure 2A). Strains contained Sgs1 and/or Rmi1 fused to an auxin-inducible degron (AID) and OsTIR1, a rice-derived, auxin-specific F-box protein expressed from a strong constitutive promoter (see Table 1). Similar strains containing a Top3-AID fusion did not display consistent Top3 depletion and therefore were not further studied (H. Kaur, unpublished data). Strains also contained a deletion of *NDT80*. Ndt80 drives meiotic expression of the Cdc5 polo-like kinase (Chu and Herskowitz 1998), which activates JM resolution in both meiotic and mitotic cells (Clyne *et al.* 2003; Sourirajan and Lichten 2008; Matos *et al.* 2011; Blanco *et al.* 2014). Thus, in ndt80*∆* cells, JMs form, but the vast majority are not resolved.

In experiments performed here, cells underwent meiosis for 6 hr and accumulated unresolved JMs in the presence of normal STR complex function. RTG was then initiated by shifting cells from sporulation medium to rich growth medium. Under these conditions, cells rapidly reduce meiotic transcripts, disassemble the synaptonemal complex, repair remaining DSBs, and resume the mitotic cell cycle, including bud emergence and a mitotic cell division (segregating sister chromatids) without an intervening S phase (Zenvirth *et al.* 1997; Friedlander *et al.* 2006; Dayani *et al.* 2011). To trigger degradation of Sgs1 and/or Rmi1, auxin was added at the time that cells were shifted to growth medium (Figure 2A). Sgs1-AID levels reduced to background (∼10% of initial levels) by 1 hr after RTG (Figure 2, B and D). Rmi1-AID depletion was less rapid, reaching ∼75% of initial levels after 1 hr, and background levels (∼10% of initial levels) at 2 hr (Figure 2, C and D). This corresponds to the time when buds first emerge and is more that 30 min before the time that the nuclear division is first visible (see Figure 4, below).

### Rmi1 and Sgs1 are needed for timely JM resolution during RTG

**Figure 3:**
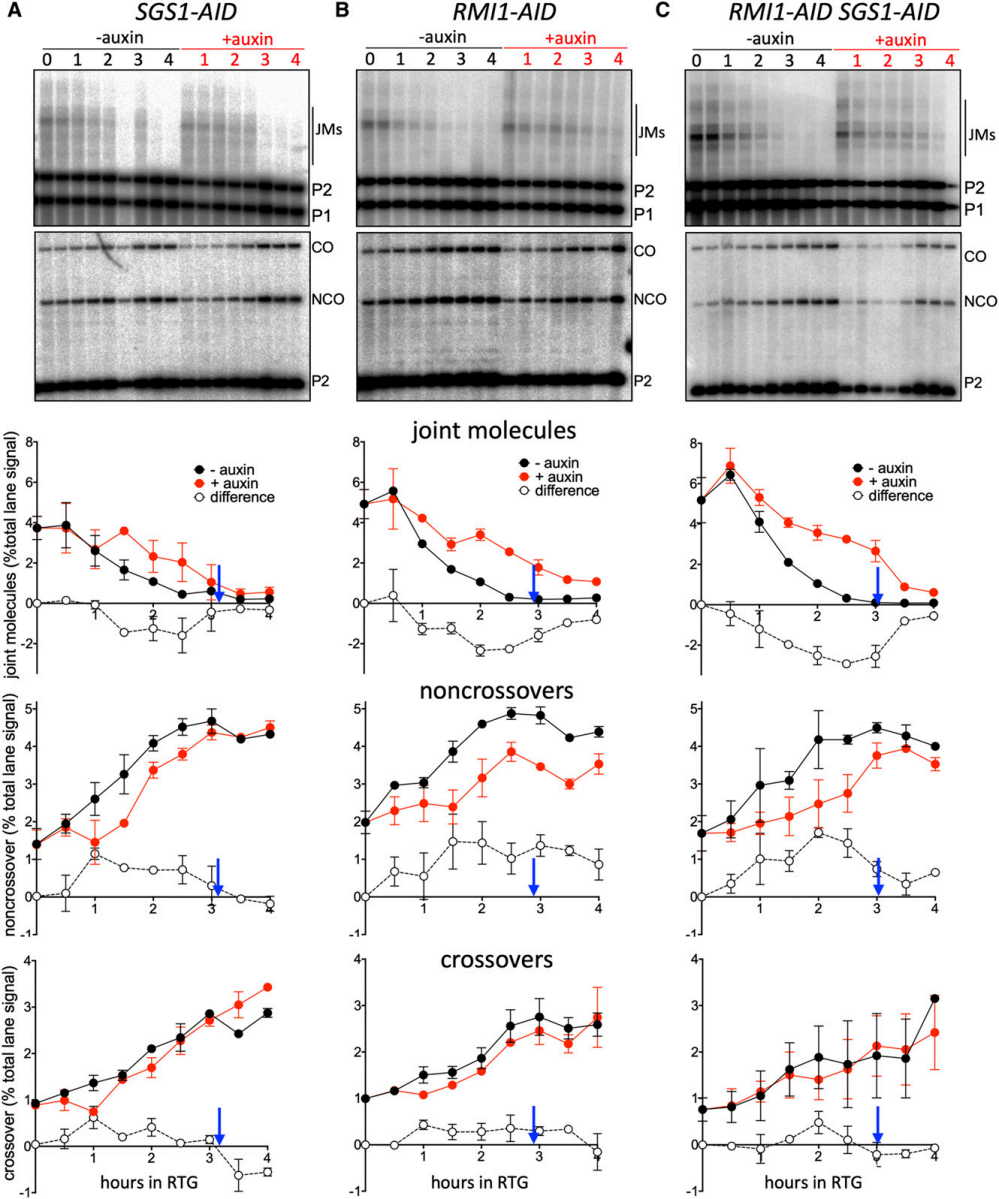
Recombination intermediate resolution and recombination product formation during RTG. DNA was extracted at the indicated times and displayed on Southern blots, using restriction enzymes and probes to detect recombination intermediates (joint molecules, JMs) or crossover (CO) and noncrossover (NCO) recombinants. For details, see Figure S1 (https://doi.org/10.25386/genetics.9841316) and *Materials and Methods*. (A) *SGS1**-AID* (MJL3807). Top two panels: representative Southern blots with *Xmn*I and *Eco*RI/*Xho*I digests, probed to detect joint molecules and recombination products, respectively. Bottom three panels: quantification of JMs, NCOs, and COs, expressed as percent of total lane signal. Red, auxin added; black, vehicle only; open circles, difference between levels when Sgs1 is present (–auxin) and depleted (+auxin). Blue arrows indicate when 50% of control cultures (–auxin) had initiated mitosis (see Figure 4, below). (B) *RMI1**-AID* (MJL3847) Details as in (A). (C) *SGS1*-*AID* *RMI1**-AID* (MJL3863). Details as in (A). Values are the mean of two independent experiments; error bars indicate range.

**Figure 4:**
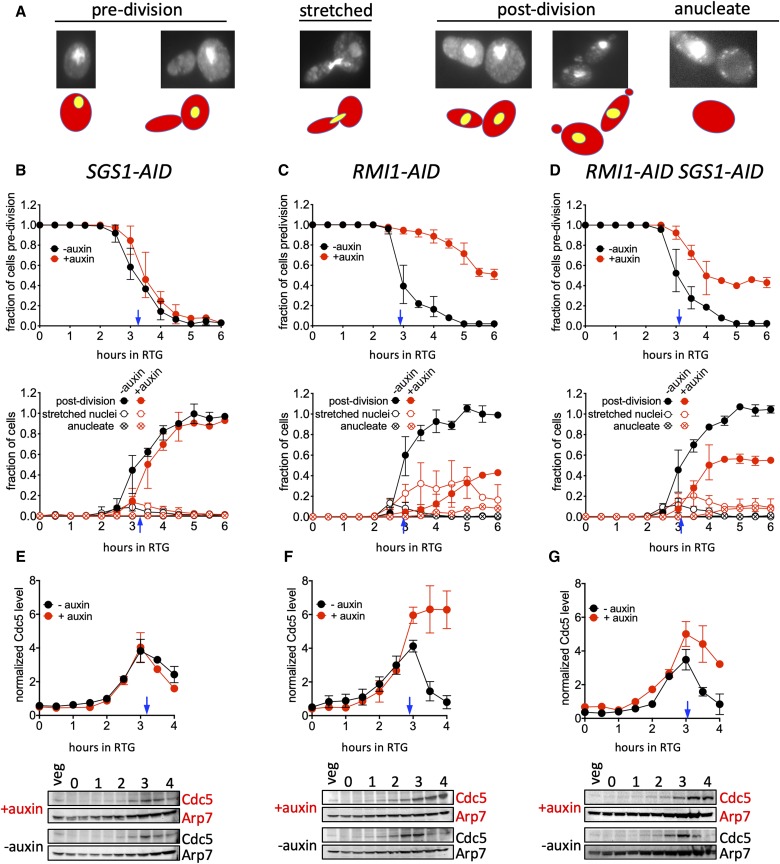
Rmi1-depletion impairs cell cycle progression during RTG. (A) Illustration of cell cycle stages, scored using fixed, DAPI-stained cells. Note that the elongated shape of the first bud to emerge during RTG allows distinction between original mother cells and daughter cells (Dayani *et al.* 2011). “Predivision”, unbudded cells and cells with a bud and a single nucleus in either the mother or daughter; “stretched”, cells with a single nucleus stretched between mother and daughter; “postdivision”, elongated, nucleated cells, with or without a bud; “anucleate”, cells with no nuclear DNA staining but with visible mitochondrial staining. Since the first division after RTG produces one elongated and one round cell, the number of elongated cells can be used to infer the number of round cells produced by this division. (B–D) Upper panel, fraction of predivision cells; lower panel cells completing mitosis (“postdivision”, solid circles) or in the midst of mitosis (“stretched”, hollow circles) for *SGS1**-AID* (MJL3807), *RMI1**-AID* (MJL3847), and *SGS1**-AID* *RMI1**-AID* (MJL3863), respectively, in control (black) or auxin-mediated depletion (red) conditions. Values from 0 to 4 hr are the mean of three independent experiments; those from 4.5 to 6 hr are the mean of two of these three experiments. Error bars indicate range. (E–G) Cdc5 protein levels during RTG in *SGS1**-AID* (MJL3807), *RMI1**-AID* (MJL3847) and *SGS1*-*AID* *RMI1**-AID* (MJL3863), respectively. Bottom panels: Representative Western blot sections probed for Cdc5 or for Arp7 as a loading control; a sample from an exponentially-growing culture (“veg”) is included to allow blot-to-blot normalization. Top panels: Normalized Cdc5 levels, calculated as the Cdc5/Arp7 ratio for experimental time points divided by the Cdc5/Arp7 ratio of the “veg” control. Blue arrows indicate when 50% of control (-auxin) cultures had initiated mitosis. Values are the mean of two independent experiments; error bars indicate range.

To monitor JM processing and resolution during RTG, we used a well-characterized recombination-reporter construct in which JMs, COs, and NCOs can be quantitatively scored on Southern blots (Jessop *et al.* 2005; Figure S1, available at https://doi.org/10.25386/genetics.9841316). Depletion of Sgs1 during RTG delayed JM disappearance and NCO formation by 1 hr relative to undepleted controls (Figure 3A), confirming previous conclusions that Sgs1 is needed for timely JM resolution during RTG (Dayani *et al.* 2011). Despite this delay, the majority of JMs had disappeared by 3.5–4 hr after RTG (12 ± 4% JMs remaining in Sgs1-depleted cells *vs.* 5 ± 2% in undepleted controls, average of 3.5 and 4 hr ± SD), when most cells had initiated mitosis (Figure 4B), and equivalent final NCO levels were achieved in both conditions. In contrast, depletion of Rmi1 during RTG both delayed and reduced JM resolution and NCO formation. Considerably more JMs remained in Rmi1-depleted cells (21 ± 4% *vs.* 4 ± 2% in undepleted controls) than in Sgs1-depleted cells (*P* = 0.014, Welch’s *t*-test). NCOs were similarly reduced, by 25 ± 8% relative to undepleted controls (Figure 3B). These findings indicate that, when Sgs1 is present, Rmi1 is important for timely JM processing and NCO formation.

Chronic loss of Top3 or Rmi1 results in a slow-growth phenotype that is suppressed in *sgs1* loss-of-function mutants (Gangloff *et al.* 1994; Chang *et al.* 2005). To see if the JM resolution and NCO formation defects observed upon Rmi1 depletion are similarly suppressed, we performed RTG experiments in which both Rmi1 and Sgs1 were auxin-depleted (Figure 3C). Sgs1 codepletion partially suppressed Rmi1 depletion phenotypes. The fraction of JMs unresolved at 3.5–4 hr was indistinguishable from those in Sgs1 depletion alone (12 ± 3% *vs.* 12 ± 4%), but JM disappearance was slower, with a partial defect in NCO formation. Final NCO levels in Sgs1/Rmi1 codepleted strains (13 ± 7% reduced relative to undepleted controls) were intermediate between Sgs1-depletion alone (0 ± 9%) and Rmi1-depletion alone (25 ± 8%). Possible reasons for this intermediate phenotype will be discussed below.

### Rmi1 depletion causes DNA segregation and cell cycle-progression defects during RTG

Unresolved JMs formed during meiosis impede chromosome segregation without affecting other steps of meiotic progression, such as spindle assembly/disassembly and spore wall formation (Jessop and Lichten 2008; Oh *et al.* 2008; De Muyt *et al.* 2012; Kaur *et al.* 2015; Tang *et al.* 2015). To see whether similar defects occur during RTG of Rmi1-depleted cells, we monitored nuclear divisions (Figure 4, A and B) in the same cultures, taking advantage of the fact that the first cell cycle after RTG, unlike subsequent cell cycles, produces elongated buds and daughter cells (Dayani *et al.* 2011). Cells were scored as predivision (either round unbudded or round mother with an elongated bud and a single nucleus in the mother), as postdivision (elongated cells containing a single nucleus), or as in metaphase or anaphase (an undivided nucleus either in the bud neck or stretched between a round mother and elongated daughter; hereafter referred to as “stretched”).

In control cultures, cells undergoing mitosis were first seen at 2.5 hr after RTG, and virtually all cells had completed mitosis by 5 hr, with only a small fraction in metaphase/anaphase at any given time (Figure 4, B–D). Cultures depleted for Sgs1 alone also initiated and completed mitosis in a timely manner, albeit with a slight delay (Figure 4B). In contrast, most Rmi1-depleted cells failed to complete mitosis (Figure 4C). A substantial fraction of Rmi1-depleted cells contained “stretched” nuclei at 5 hr after RTG, a time when mitosis was complete in control cultures. Upon further incubation, this fraction declined, and postdivision cells lacking a nucleus (anucleate cells) appeared at low levels. Cultures codepleted for Sgs1 and Rmi1 displayed an intermediate phenotype (Figure 4D). About half appeared to execute mitosis with timing similar to controls, while the rest failed to divide. As in Rmi1-depleted cultures, a substantial fraction of cells that failed to divide contained “stretched” nuclei, and anucleate cells appeared at low levels upon continued outgrowth. This mixed phenotype parallels the partial defects seen in molecular analyses (Figure 3C, above). Together, the cytological and molecular phenotypes of Sgs1/Rmi1 codepleted cells suggests that these cultures are heterogeneous, with Rmi1 depletion-induced defects being suppressed in only about half of cells.

### Progression defects in Rmi1-depleted cells are due to a cell cycle arrest

We considered two possible reasons for the failure of Rmi1-depleted cells to complete the first mitosis after RTG. The first is that a mechanical barrier, created by unresolved JMs, prevents nuclear division. If this were the case, cells would be expected to progress through mitosis, but might not divide chromosomes between mother and daughter cells. Alternatively, it is possible that unresolved JMs or other DNA structures, formed in the absence of Rmi1, are recognized by a checkpoint system that prevents cell cycle progression.

As an initial test, we monitored levels of the Cdc5 polo-like kinase, which is required for full SSN activity during late G2 and mitosis (Matos *et al.* 2011; 2013). Cdc5 is produced during G2/M (Cho *et al.* 1998), and is degraded upon exit from mitosis (Visintin *et al.* 2008). Cdc5 was first detectable at 1.5–2 hr after initiation of RTG. In control cultures and Sgs1-depleted cultures, Cdc5 accumulated until ∼3 hr, when about half of the cells had initiated mitosis. Cdc5 levels then declined, consistent with these cells exiting mitosis and initiating a second cell cycle (Figure 4, E–G). In contrast, in Rmi1-depleted cultures, Cdc5 accumulated to greater levels and never declined (Figure 4F), consistent with a block before exit from mitosis. In cultures that were doubly depleted for Sgs1 and Rmi1, Cdc5 accumulated and then declined, but did not decline to the same extent as in control cultures (Figure 4G), consistent with the previous inference of culture heterogeneity. Because of the more profound effects seen in Rmi1-depleted cultures, and because of the complications inherent in the analysis of heterogeneous cultures, we focused further efforts on characterizing the arrest seen with Rmi1-depletion alone.

**Figure 5:**
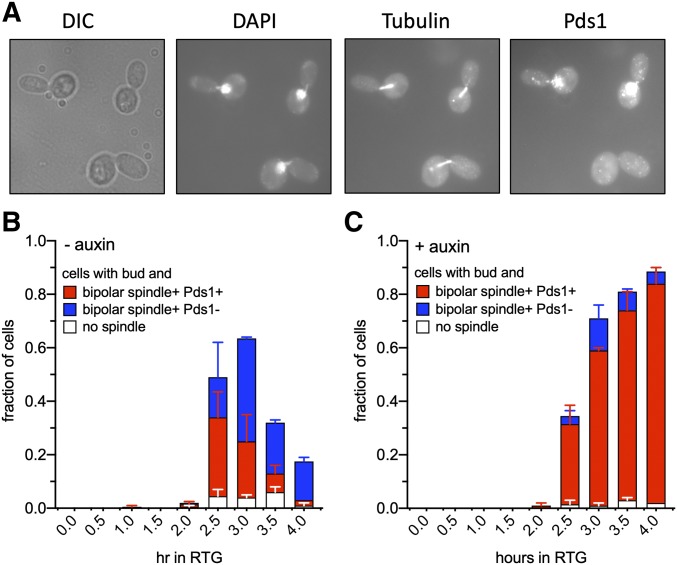
Rmi1-depletion causes a G2/M arrest during RTG. (A) Three Rmi1-depleted mother-daughter cell pairs from an auxin-treated *RMI1**-AID* culture (MJL3899) taken 4 hr after shift to rich medium containing auxin. From left to right, differential interference contrast image, detection of DNA (DAPI), beta-tubulin, and Pds1-Myc. See *Materials and Methods* for details. The bottom mother-daughter pair was scored as having undergone the metaphase–anaphase transition, based on the absence of Pds1. (B) Percent of total cells in a control culture with a bud and lacking a bipolar spindle (white) or containing a bipolar spindle and nuclear Pds1 (red) or with a bipolar spindle but lacking nuclear Pds1 (blue). (C) As in (B), but in the presence of auxin. Data are from two experiments, error bars denote range.

To further characterize this arrest, we monitored spindle morphology and levels of Pds1, the budding yeast securin (Figure 5). Pds1 accumulates in nuclei during G2 and metaphase, and is degraded at the metaphase-anaphase transition (Cohen-Fix *et al.* 1996). Control cultures displayed all the hallmarks of cells progressing unimpeded through mitosis, including bud formation, a transition from G2/metaphase (cells with bipolar spindles and intranuclear Pds1) to anaphase/post-anaphase (cells with bipolar spindles but lacking intranuclear Pds1), and mother-bud separation (Figure 5B). In contrast, Rmi1-depleted cultures rarely underwent mother-bud separation, and the vast majority of cells contained bipolar spindles and intranuclear Pds1, consistent with a G2/M cell cycle arrest (Figure 5C). Taken together, these data indicate that Rmi1 depletion during RTG results in both incomplete JM resolution and cell cycle arrest before the metaphase-anaphase transition.

### Cell cycle arrest during RTG in Rmi1-depleted cells is mediated by the DNA damage response

**Figure 6:**
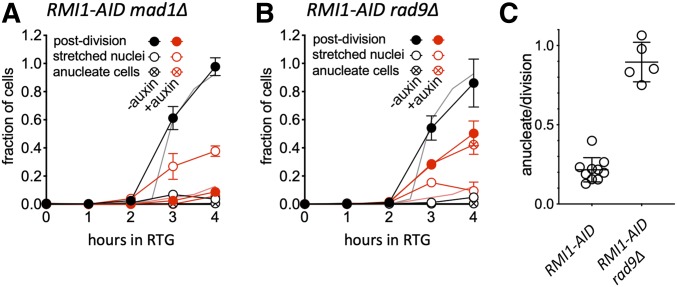
The DNA damage checkpoint is responsible for arresting cell cycle progression during RTG in the absence of Rmi1. (A) Fraction of cells completing cell division (solid circles), at metaphase/anaphase (with chromosomal DNA “stretched” between mother and daughter; hollow circles), or without nuclei (circles with cross) in spindle assembly checkpoint-defective *RMI1**-AID **mad1*∆ cells (S5338xS5344) during RTG in the absence (black) or presence (red) of auxin. (B) As in (A), but with DNA damage checkpoint-defective *RMI1**-AID **rad9*∆ diploids (S5342xS5348). In both (A) and (B), gray and pink lines without symbols are postdivision values for corresponding *MAD1* *RAD9* diploids, from Figure 4C. (C) Fraction of divisions producing an anucleate cell. Values are from the following time points of two independent experiments: *RMI1**-AID*, 4–6 hr, *RMI1**-AID **rad9*∆, 2–4 hr.

Two major cell cycle checkpoint systems block Pds1 degradation and cause a G2/M cell cycle arrest: the spindle assembly checkpoint, which detects the presence of kinetochores that are not attached to spindle microtubules (Cohen-Fix *et al.* 1996; Wells 1996); and the DNA damage checkpoint, which detects unrepaired DNA damage (Cohen-Fix and Koshland 1997; Agarwal *et al.* 2003; Harrison and Haber 2006). To determine which system blocks progression during RTG in the absence of Rmi1, we deleted either *MAD1* or *RAD9*, which are essential for the spindle assembly and DNA damage checkpoints, respectively (Figure 6). When Rmi1 was present, both *mad1*∆ and *rad9*∆ mutants underwent RTG with wild-type efficiency and kinetics, with ∼90% of cells completing nuclear and cellular division by 4 hr after RTG. Rmi1-depleted *mad1*∆ cells displayed arrest phenotypes similar to those seen in *MAD1* Rmi1-depleted cells. Only 9% of cells completed mitosis by 4 hr after RTG, and a large fraction of cells (∼40%) contained nuclei with chromosomal DNA stretched between mother and daughter (Figure 6A). In contrast, more than half (57%) of Rmi1-depleted *rad9*∆ mutant cells completed the first cell division after RTG (Figure 6B), as compared to 8% of undepleted cells. Thus, the DNA damage checkpoint is largely responsible for the observed cell cycle arrest.

While *rad9*∆ restored cell cycle progression to Rmi1-depleted cells, it did not restore normal chromosome segregation. Instead, at 4 hr after RTG, ∼40% of cells lacked a detectable nucleus (Figure 6B). This corresponds to ∼90% of divisions producing a cell (either mother or daughter) without a nucleus (Figure 6C). This stands in contrast to the much lower level of anucleate cells produced in *RAD9* Rmi1-depleted cultures (20% of divisions, Figure 4C), where the majority of cells remained arrested. It suggests that unresolved recombination intermediates are present in Rmi1-depleted cells at levels sufficient to mechanically block chromosome segregation when the arrest is bypassed in *rad9*∆ mutants.

## Discussion

### STR-mediated dissolution is an important resolution mechanism during RTG

**Figure 7:**
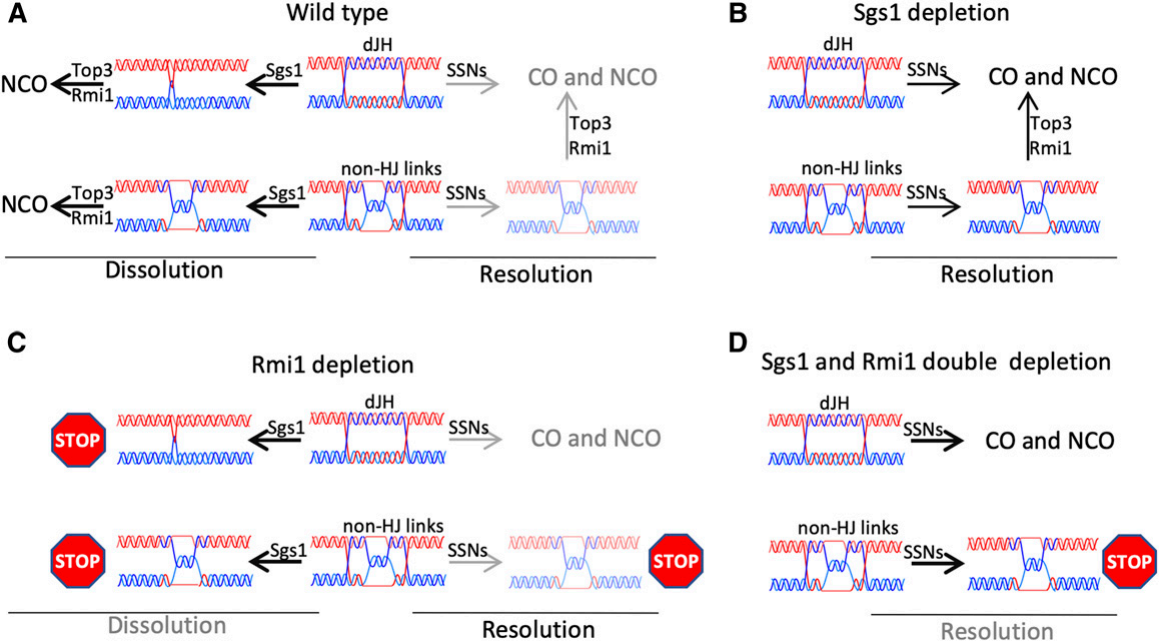
Recombination intermediate resolution during RTG. (A) In cells where the STR is fully functional, most recombination intermediates are resolved by convergent branch migration, shown as involving Sgs1, but which also may involve Top3-Rmi1. The resulting molecules are proposed to contain single-strand interlinks (hemicatenanes), which Top3-Rmi1 resolves to form noncrossovers (NCOs). A minor fraction of recombination intermediates escape Sgs1 activity and are processed by Holliday junction-resolving nucleases (SSNs). Those that contain only Holliday junctions (top row) are fully resolved, while those containing both Holliday junctions and other strand interlinks (here illustrated as hemicatenanes) require both SSNs and Top3-Rmi1 to produce fully resolved products. (B) In the absence of Sgs1, Holliday junctions in recombination intermediates can be cleaved by SSNs, but intermediates that contain non-HJ interlinks require Top3-Rmi1 to be fully resolved. (C) In the absence of Rmi1, Sgs1-catalyzed convergent branch migration produces molecules with structures that cannot be further resolved, and which induce a Rad9-dependent cell cycle arrest. As in wild-type, some intermediates escape Sgs1 activity and are resolved by SSNs; those containing non-HJ interlinks remain unresolved and, upon stretching, the ssDNA they contain contributes to the cell cycle arrest. (D) In the absence of both Sgs1 and Rmi1, recombination intermediates that contain only Holliday junctions can still be efficiently resolved, while intermediates that also contain non-HJ interlinks will remain unresolved and induce a cell cycle arrest. This figure ignores the possibility that non-STR activities also may contribute to branch migration and decatenation.

Two *in vitro* activities of Sgs1/BLM-Top3-Rmi1, D-loop disassembly (van Brabant *et al.* 2000; Bachrati *et al.* 2006; Fasching *et al.* 2015) and dHJ dissolution (Wu and Hickson 2003; Plank *et al.* 2006; Wu *et al.* 2006), can potentially limit JM and CO accumulation. Most *in vivo* studies have scored either CO end-products or steady-state JM levels, and thus could not distinguish STR/BTR preventing dHJ-JM formation from STR/BTR driving dHJ-JM resolution as NCOs. In the current study, we focused directly on dHJ resolution during RTG under conditions of Sgs1 and/or Rmi1 depletion. Our findings indicate that STR-mediated dissolution is an important mode for dHJ-JM resolution *in vivo*, and that Top3-Rmi1 has important STR-independent functions (see Figure 7).

dHJ-JMs can be resolved during the mitotic cell cycle either by dissolution or by SSN-mediated cleavage; only the latter can produce COs (Matos and West 2014). We find that, when STR is active, most JM resolution precedes Cdc5 expression and thus SSN activation (Figure 2 and Figure 3). Moreover, during RTG, Sgs1, or Rmi1 depletion markedly delays JM resolution and NCO formation without changing the time or levels of CO formation (Figure 3). Thus, our findings are consistent with STR-mediated dHJ dissolution being the primary mode of JM resolution during RTG, and, therefore, during the mitotic cell cycle.

Still remaining to be answered is the question of how JMs are resolved when STR components are depleted. In the absence of STR activity, JMs not resolved by dissolution should be resolved by SSN-mediated cutting in late G2 and mitosis, and thus should produce fewer NCOs and more COs. Our data only partially support this expectation, as NCO formation is delayed when Sgs1 and/or Rmi1 are depleted (Figure 3), but COs do not increase. This may be because auxin-mediated depletion did not completely eliminate STR activity, and the remaining active fraction might have resolved JMs by dissolution before SSNs are activated. Alternatively, when Sgs1 was depleted, Top3-Rmi1, either by itself or in combination with other helicases, might have catalyzed JM dissolution in the absence of Sgs1, as has been reported for D-loop disassembly *in vitro* and *in vivo* (Fasching *et al.* 2015; Piazza *et al.* 2019).

### Rmi1 is required for full JM resolution and cell cycle progression during RTG

Previous studies suggest that Top3-Rmi1 has Sgs1-independent functions during the mitotic cell cycle and during meiosis, to limit accumulation of JMs that cannot be resolved by standard HJ resolvases (Wallis *et al.* 1989; Gangloff *et al.* 1994; Chang *et al.* 2005; Mullen *et al.* 2005; Kaur *et al.* 2015; Tang *et al.* 2015). We found that, when Rmi1 was depleted during RTG, unresolved JMs remained even when Cdc5 levels were high and SSN resolvases should have been fully activated (Figure 4). This is consistent with the suggestion that Top3-Rmi1 removes intermolecular DNA connections that cannot be cleaved by HJ resolvases during the mitotic cell cycle, as it does during meiosis (Kaur *et al.* 2015; Tang *et al.* 2015).

The suppression of *top3* and *rmi1* slow-growth phenotypes by *sgs1* or recombination mutants has led to the suggestion that toxic recombination intermediates are formed by Sgs1 when Top3-Rmi1 is absent (Gangloff *et al.* 1994; Oakley *et al.* 2002; Chang *et al.* 2005; Shor *et al.* 2005). In our study, Sgs1 codepletion only partially suppressed Rmi1 depletion-associated defects (Figure 3C and Figure 4D). This might have been due to residual Sgs1 activity, possibly present in only some of the cells in the population. However, it is also possible that, even in the complete absence of Sgs1, “toxic” JMs are formed during normal meiosis that require Top3-Rmi1 for their resolution (cf. Tang *et al.* 2015). Persistence of these JMs In Sgs1/Rmi1 codepleted cells, possibly at levels that vary from cell to cell, might explain the heterogeneous phenotypes of Sgs1/Rmi1 codepleted cells. Regardless of which explanation is correct, the more penetrant defects observed when Rmi1 alone is depleted support previous suggestions that Sgs1 activity creates JMs that require Top3-Rmi1 for their resolution.

### A DNA damage response-dependent cell cycle arrest during RTG in the absence of Rmi1

Rmi1 depletion during RTG causes a cell cycle arrest at the metaphase–anaphase transition (Figure 4C and Figure 5C) that is bypassed by *rad9*∆, indicating that it is due to the DNA damage checkpoint (Figure 6B). Remarkably, when this checkpoint is bypassed, almost all of the cells that progress produce an anucleate cell, consistent with unresolved JMs preventing bulk chromosome segregation. We estimate that, in Rmi1-depleted cells, ∼40% of segregating chromatid pairs are linked by unresolved JMs (see *Materials and Methods*). While this might not be enough to completely block chromosome segregation, the above estimate is based on frequencies of JMs that migrate as discrete species in gels (Figure 3 and Figure S1). The persistent lane background seen in Rmi1-depleted cultures (Figure 3B) may reflect the presence of additional unresolved intermediates that might have contributed additional interchromatid connections. Further studies will be required to determine the precise nature of these segregation-blocking connections, and of the structures that induce the *RAD9*-dependent DNA-damage checkpoint. Consistent with our finding of a DNA damage checkpoint induced by unresolved JMs during RTG, previous studies have shown that *top3* and *rmi1* mutants display many hallmarks of low-level activation of the DNA damage response (Gangloff *et al.* 1994; Chakraverty *et al.* 2001; Chang *et al.* 2005). Evidence for a DNA damage checkpoint induced by unresolved recombination intermediates is also provided by a report that sgs1∆ *mms4*-*14A* and sgs1∆ *cdc5*-*2* mutant strains, which do not activate the Mus81-Mms4 resolvase, also contain an elevated fraction of cells in G2/M (Matos *et al.* 2013).

The DNA damage response is initiated when the Mec1-Ddc2 (ATR-ATRIP) checkpoint kinase interacts with replication protein A-coated single stranded DNA present at unrepaired DNA lesions; Mec1 then acts through intermediary sensors and effectors, including Rad9, to cause cell cycle arrest (reviewed in Nyberg *et al.* 2002). How might unresolved recombination intermediates present during RTG activate the DNA damage response? Little, if any, break-associated single-strand DNA is expected to be present, since most meiotic DSBs are repaired before the shift to rich medium, especially in ndt80∆ cells, and the few DSBs that remain are rapidly repaired after RTG (Dayani *et al.* 2011).

We suggest that the DNA damage response is induced during RMI-depleted RTG, or in sgs1∆ cells unable to activate Mus81-Mms4 (Matos *et al.* 2013), when unresolved intermediates are stretched by the mitotic spindle and expose single-strand DNA (Figure 7). This in turn raises the question of why similar behavior is not seen during budding yeast meiosis, where meiotic divisions proceed in the presence of unresolved JMs (Jessop and Lichten 2008; Oh *et al.* 2008; Kaur *et al.* 2015; Tang *et al.* 2015), or during mitosis in mammalian cells, where cells with unresolved links between sister chromatids proceed to anaphase and form ultrafine DNA bridges (Chan and Hickson 2011; Chan *et al.* 2018). The answer to this question may lie in the different ways that the DNA damage checkpoint functions during the mitotic cell cycle in budding yeast on one hand, and in meiotic yeast and in mammalian cells on the other. During the budding yeast mitotic cell cycle, chromosomes are always attached to the spindle (Winey and Bloom 2012), and the DNA damage checkpoint blocks the metaphase–anaphase transition (Nyberg *et al.* 2002). Thus, spindle-mediated stretching of unresolved recombination intermediates has the potential to form checkpoint-inducing ssDNA. During budding yeast meiosis, the DNA damage checkpoint blocks expression of the Ndt80 transcription factor that is required for formation of the metaphase I spindle; thus, the meiosis I spindle does not form until cells have progressed beyond the checkpoint and are irreversibly committed to undergo meiotic divisions (Winter 2012; Subramanian and Hochwagen 2014; Tsubouchi *et al.* 2018). In a similar vein, the DNA damage response in mammalian cells primarily blocks progression before chromosomes attach to the spindle (Nyberg *et al.* 2002), and multiple mechanisms limit DNA damage response signaling once cells have entered mitosis (Heijink *et al.* 2013). In both situations, ssDNA would not form at unresolved JMs until it was too late to prevent chromatid separation. Thus, a DNA damage response-mediated cell cycle arrest provoked by unresolved recombination intermediates may be a specific feature of organisms that undergo closed mitosis, and in which chromosomes are always attached to the spindle.

### Concluding remarks

In this work, we have presented data indicating that Sgs1(BLM)-Top3-Rmi1-mediated dissolution is a predominant mechanism for recombination intermediate resolution during the mitotic cell cycle, thus providing *in vivo* confirmation of a mechanism previously proposed by *in vitro* biochemical studies. Our findings also confirm previous suggestions that, in the absence of Top3-Rmi1 decatenase activity, Sgs1 helicase creates entangled structures that cannot be resolved by Holliday junction-cleaving nucleases; similar structures may also be present, albeit at lower levels, in recombination intermediates that form when STR is fully active. Even though all DNA strands in these structures are expected to be intact, our data suggests that their presence activates the DNA damage checkpoint. This unresolved recombination intermediate checkpoint, which perhaps is unique to the budding yeast cell cycle, may be responsible for the observed recombination- and Sgs1-dependent slow growth and G2/M accumulation of *top3* and *rmi1* mutants, and will be fertile ground for future investigation.
